# Update on Long-Acting Anticholinergics in Children and Adolescents With Difficult and Severe Asthma

**DOI:** 10.3389/fped.2022.896865

**Published:** 2022-07-19

**Authors:** Francesca Santamaria, Carla Ziello, Paola Lorello, Cristina Bouchè, Melissa Borrelli

**Affiliations:** Department of Translational Medical Sciences, University of Naples Federico II, Naples, Italy

**Keywords:** asthma, children, adolescents, preschool children, long-acting anticholinergics, tiotropium bromide

## Abstract

Tiotropium bromide is the only long-acting muscarinic antagonist (LAMA) approved for treatment of patients aged ≥6 years old who have symptoms of uncontrolled asthma. Results from several clinical trials have found that once-daily inhaled tiotropium bromide is safe and efficacious in 6- to 17-year-olds with symptomatic asthma despite treatment with inhaled corticosteroids, with or without other medications. There are still few available studies investigating the impact of tiotropium bromide treatment in preschool children with suboptimal control. In this narrative review, we summarize the pharmacological effects of the LAMA tiotropium bromide, provide an overview about current asthma studies at different pediatric ages, and describe future research needs.

## Introduction

Asthma is a multifactorial inflammatory disorder of the airways that in 2019 affected approximately 262 million people and caused 461,000 deaths (1), with estimates that 400 million people will be affected by 2025 (2). Currently, asthma is also the most common chronic non-communicable pediatric disease worldwide (3). Exacerbations due to allergen or respiratory pathogens exposure, or exercise-induced are the main causes of hospitalization among children or adolescents with asthma, and this in turn results in schoolchildren and work-parents’ absenteeism and high direct and indirect health care costs (4).

In recent years, it has been emphasized that for improving health status the overall goal of asthma management is to achieve symptom control rather than assessing patients based on symptom severity (5). Lack of control in asthma includes persistence of clinical symptoms, high number of exacerbations requiring rescue medications, and progressive lung function deterioration. The importance of symptom control in children is underscored by the results of a national survey that found that asthma control fell short on nearly every goal, indicating a lack of effective asthma symptom control in affected children (6). Moreover, a great proportion of patients with even mild symptoms are inadequately controlled and may face severe exacerbations (7).

Reasons for suboptimal control in children and adolescents include incorrect diagnosis of asthma, especially when spirometry cannot be accurately obtained by uncooperating patients or young children; persistent exposure to environmental triggers such as tobacco smoke or allergens; low or non-adherence to treatment and/or poor inhaler techniques, which are frequently described when long-term anti-inflammatory medications are prescribed; personal concerns about potential adverse effects; evidence of comorbidities, for instance gastroesophageal reflux, obesity, rhinitis, and/or recurrent airway infections (8). Therefore, based on all the above observations, physicians dealing with asthma feel the strong need to have alternative therapeutic interventions available for patients with uncontrolled asthma.

Patients with asthma are now recommended to take inhaled corticosteroids (ICS) whenever given short-acting β2-agonists (SABA) as rescue therapy (9). This is supported by the evidence that ICS enhance the expression of β*2*-adrenergic receptors in the airways, prevent severe exacerbations and maintain symptoms control (10). Indeed, according to the Global Initiative for Asthma (GINA) and National Asthma Education and Prevention Program (NAEPP), low-dose ICS is recommended as the best initial treatment when asthma symptoms are under suboptimal control (9, 11). At GINA Steps 3–4 and at NAEPP Steps 4–5, the ICS/Long-Acting beta-agonists (LABA) combination is recommended and in case of further lack of response add-on alternative options are suggested (9, 11). Finally, patients at any age who have persistent symptoms or experience exacerbations despite good adherence to Step 4 treatment and in whom other controllers have been previously considered, should be referred to a center specialized in the management of severe asthma for treatment optimization, i.e., re-evaluation of diagnosis, modification of ongoing therapy or addition of other medications (9, 11, 12).

According to the most recent definitions from adult and pediatric literature, difficult-to-treat asthma is characterized by symptoms that persist despite ICS–LABA treatment even at high-dose ICS, while severe asthma is asthma that is uncontrolled despite good adherence with high-dose ICS– LABA and management of comorbidities, or that worsens when high-dose treatment is decreased (9, 11).

It is indeed important to identify any modifiable factors to differentiate children with difficult asthma from those with true severe therapy-resistant asthma. Acting early on modifiable factors in children with difficult asthma allows better control of symptoms without further investigations. In the absence of these factors, addressing a correct diagnosis of true therapy-resistant severe asthma avoids diagnostic and therapeutic delays, allowing patients to benefit from the use of new therapies (13).

In the last decades, the use of long-acting muscarinic antagonists (LAMA) including tiotropium bromide, glycopyrronium, and umeclidinium as bronchodilators in the long-term treatment of asthma has progressively increased (14). The LAMA tiotropium bromide has recently been incorporated into the GINA document at Steps 4 and 5 in patients with a history of exacerbations (9). However, GINA experts recommend that, in patients experiencing exacerbations despite low-dose ICS/LABA, the ICS dose should be increased to at least medium dose, or treatment converted to Maintenance and Reliever Therapy (MART) with ICS/formoterol before considering the addition of a LAMA (9). In individuals aged 12 years and older with persistent asthma that is not controlled by ICS therapy alone, the NAEPP Expert Panel recommends adding a LABA rather than a LAMA to an ICS (11). However, if the individual is not using or cannot use LABA therapy, adding a LAMA to an ICS is indicated as an acceptable alternative.

New treatment options for asthma are indeed strongly needed in children or adolescents with asthma, especially those with moderate or severe symptoms (15). In the last decade, several pediatric studies have evaluated the use of tiotropium bromide as an add-on to ICS maintenance therapy, with or without leukotriene receptor antagonist (LTRA) or LABA. This narrative review will summarize the pharmacological effects of the LAMA tiotropium bromide, provide an overview about current asthma studies at different pediatric ages, and describe future research needs.

## Pharmacologic Characteristics of Tiotropium Bromide and Its Administration

Acetylcholine is a neurotransmitter which is released from the neurons of the parasympathetic nervous system in several tissues, including the lung. Acetylcholine stimulates smooth muscle contraction and mucus secretion through M1 to M5 muscarinic receptors (16).

Beyond bronchoconstriction, acetylcholine also regulates airway inflammation and remodeling (17).

Anticholinergic agents are antiasthma medications. Initially, most of the literature was focused on the use of the short-acting anticholinergic ipratropium bromide, a medication predominantly used in combination with SABA to treat bronchoconstriction during asthma exacerbations (18). In 1989, the tiotropium bromide (bromide salt) was patented and then approved for medical use in the form of inhalation powder in 2002 as LAMA bronchodilator drug (19).

**FIGURE 1:**
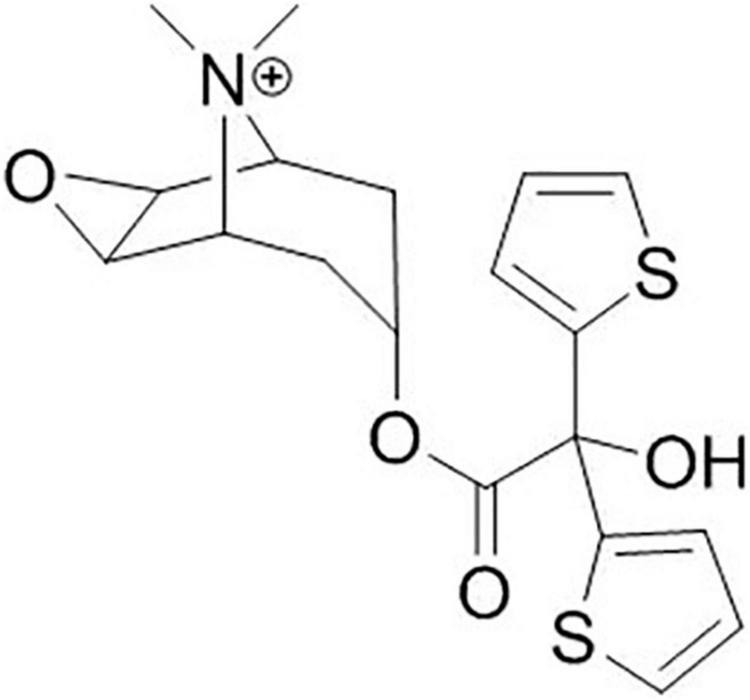
Moulecular structure of tiotropium.

Tiotropium bromide is a quaternary ammonium derivative, structurally related to ipratropium bromide (Figure 1), but with a significantly higher affinity for muscarinic receptors within the airways. Tiotropium bromide reversibly binds to the M1, M2, and M3 receptors of the airway smooth muscles, and blocks the effects of the acetylcholine released by parasympathetic nerve endings through a competitive and reversible inhibition, with faster dissociation rates from M2 than from M1 or M3 receptors (20). Tiotropium bromide has a maximum effect occurring at 30–60 min, and since the cholinergic transmission is blocked approximately for 35 h, its principal anti-asthmatic property is the long-acting bronchodilation, which allows a once-daily administration.

A pharmacokinetic study in children aged 6–11 years old demonstrated that tiotropium bromide is rapidly absorbed following inhalation and then excreted into urine (21), confirming that systemic exposure of children to the medication is within the range observed in adults (22).

**FIGURE 2:**
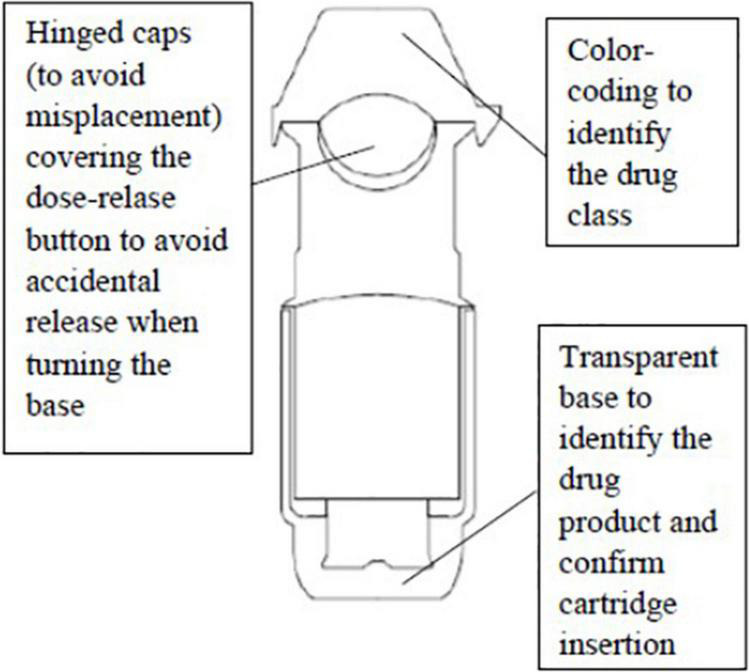
Externarl structure of Respimat© inhaler.

**FIGURE 3:**
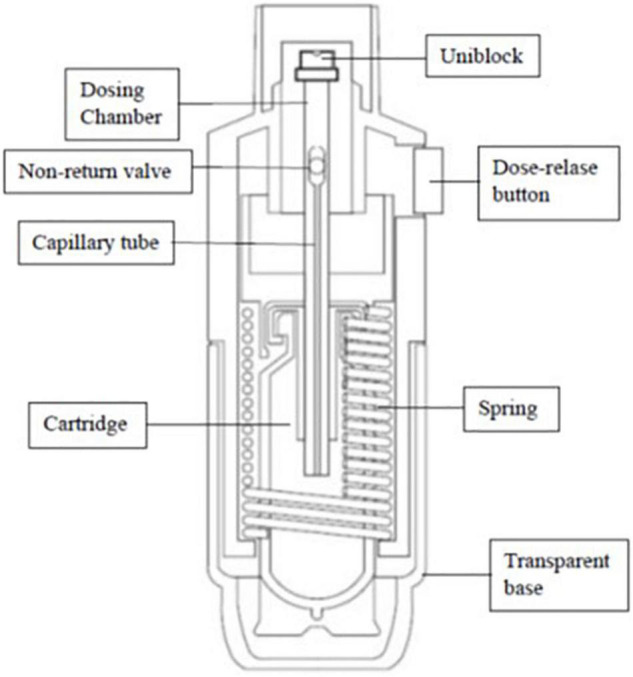
Internal structure of Respimat© inhaler.

Tiotropium bromide is administered through the Respimat^©^ inhaler (19) (Figures 2, 3). Most children aged ≥6 years can use a Respimat^©^ inhaler without a valved spacer device (23) but younger children should use the Respimat in combination with a valved spacer (24, 25).

## Evidence of Tiotropium Bromide Use in the Pediatric Population

Unlike inhaled short acting anticholinergic ipratropium bromide that has been extensively investigated in children or adolescents with acute asthma (26), LAMA has been less studied and pediatric studies regarding the use in chronic asthma only date back to the last 2 decades (Table 1).

**TABLE 1:** Overview of published studies of tiotropium bromide in pediatric patients with asthma.

Author	Study design	Age group (years)	Asthma severity	Daily dose and treatment duration	Primary outcome	Main findings
					Peak FEV1 (0–3 h):	
Vogelberg et al. (28)	Phase 2, double-blind, placebo-controlled, dose- ranging, incomplete crossover study	Adolescents (12–17)	Moderate persistent asthma	5 μg 2.5 μg 1.25 μg	5 μg: 113 mL *p* = 0.004 2.5 μg: 57 mL *p* = 0.148	Improvement of lung function vs. placebo. Safe and well tolerated
				12 weeks	1.25 μg: 67 mL	
					*p* = 0.066	
					Peak FEV1 (0–3 h):	
Vogelberg et al. (33)	Phase 2, double-blind, placebo-controlled, incomplete-crossover, dose-ranging study	School-age children (6–11)	Symptomatic asthma	5 μg 2.5 μg 1.25 μg 12 weeks	5 μg: 87 mL *p* < 0.0002 2.5 μg: 104 mL *p* < 0.0001 1.25 μg: 75 mL *p* = 0.001	Improvement of lung function vs. placebo. Safe and well tolerated
					Peak FEV1 (0–3 h):	
Hamelmann et al. (29)	Phase 3, double-blind, placebo-controlled, parallel-group trial	Adolescents (12–17)	Moderate symptomatic asthma	5 μg 2.5 μg 48 weeks	5 μg: 174 mL (95% CI, 76–272 mL) *p* < 0.001 2.5 μg: 134 mL (95% CI, 34–234 mL) *p* < 0.01	Improvement of lung function vs. placebo. Safe and well tolerated
Huang et al. (36)	Phase 3, double-blind, placebo-controlled study	School-age children and adolescents (6–14)	Moderate symptomatic asthma	1.25 μg 12 weeks	FEV1% at week 12 and FVC at week 8 *p* < 0.05. Other indicators *p* < 0.01	Improvement of lung function vs. placebo. Decreased need for SABA Night symptoms improvement
					Peak FEV1 (0–3 h):	
Hamelmann et al. (30)	Phase 3, double-blind, placebo-controlled, parallel-group study	Adolescents (12–17)	Severe symptomatic asthma	5 μg 2.5 μg 12 weeks	5 μg: 90 mL (95% CI, –19 –198 mL) *p* = 0.104 2.5 μg:111 mL (95% CI, 2–220 mL) *p* = 0.046	Improvement of lung function only at 2.5 μg vs. placebo.
					Peak FEV1 (0–3 h):	
Szefler et al. (34)	Phase 3, double-blind, placebo-controlled, parallel-group study	School-age children (6–11)	Severe symptomatic asthma	5 μg 2.5 μg 12 weeks	5 μg: 139 mL (95% CI, 75–203 mL) *p* < 0.001 2.5 μg: 35 mL (95% CI, –28 to 99 mL) *p* = 0.27	Improvement of lung function only at 2.5 μg vs. placebo. Well tolerated
					Peak FEV1 (0–3 h) (week 24)	
Vogelberg et al. (35)	Phase 3, double-blind, placebo-controlled, parallel-group study	School-age children (6–11)	Moderate symptomatic asthma	5 μg 2.5 μg 48 weeks	5 μg: 164 mL (95% CI, 103–225 mL) *p* < 0.001	Improvement of lung function vs. placebo.
					2.5 μg: 170 mL (95% CI, 108–231 mL) *p* < 0.001	
Vrijlandt et al. (41)	Phase 2/3, double-blind, placebo-controlled, parallel-group study	Pre-school children (1–5)	Persistent asthma symptoms (wheezing, cough, shortness of breath)	5 μg 2.5 μg 12 weeks	Combined daytime asthma symptom score: 5 μg: –0.048 (95% CI, –0.29 to 0.19) 2.5 μg: –0.080 (95% CI, –0.31 to 0.15)	Asthma scores not significantly different between the two active groups vs. placebo. Potential to reduce asthma exacerbations vs. placebo Tolerability as placebo

*CI, confidence interval; FEV1, forced expiratory volume in 1 second; FVC, forced vital capacity; FEV1 (0–3 h), forced expiratory volume in 1 second within 3 hours after dosing; ICS, inhaled corticosteroid; SABA, short-acting β2 agonists; LABA, long-acting β2-agonist.*

### Evidence of Tiotropium Bromide Use in Adolescents With Asthma

The effects of tiotropium bromide inhalation in the pediatric population with chronic uncontrolled asthma were first evaluated in adolescents, and results opened the road for assessing tiotropium in younger children. Most of the published studies are randomized controlled trials (RCT) and focus on type and severity of subjective clinical symptoms [evaluated by the asthma standardized questionnaires such as the Asthma Control Questionnaire (ACQ) or Asthma Control Test (ACT)], and lung function (namely, spirometry) as primary outcomes. The lung function end points included the peak expiratory flow (PEF) or the forced expiratory flow at 1 s (FEV1) either reported as peak FEV1 (within 3 h after administration of the study drug) or trough FEV1, i.e., the predose FEV1 measured at the end of the dosing interval, 10 minutes before the next dose of trial medication at week 24 or week 12 (27), or, less frequently, as forced expiratory flow at 25–75% of the lung volume (FEF25–75%). In 2014, Vogelberg conducted an incomplete crossover RCT of 105 adolescents with moderate symptomatic asthma, who were administered once-daily tiotropium (5, 2.5, and 1.25 μg) as an add-on therapy to medium-dose ICS with or without LTRA. Results showed that peak FEV_1_, trough FEV_1_ and FEV_1_ Area Under the Curve (AUC) (0–3 h) significantly improved (28). The term “incomplete” refers to the study design that requires that treatments are grouped into sets or “blocks,” not all of which include every treatment, and each block is administered to a different group of participants to avoid administering too many treatment conditions to the same group of participants.

Results from two additional phase III RCT of once- daily tiotropium administered for 12 or 48 weeks confirmed lung function beneficial effects in adolescents treated with long-term ICS with or without other controller therapies (29, 30). However, only the 5-μg dose significantly improved trough FEV1 at week 24, while in the 48-week RCT asthma control improved using both 5 and 2.5 μg doses of tiotropium, with the 2.5 μg dose also significantly reducing rescue medications use (29). The incidence of adverse effects including asthma worsening or exacerbations, decreased PEF rate, nasopharyngitis, and viral respiratory tract infection was comparable across the treatment groups and did not lead to discontinuation of treatment, as previously reported (31).

In conclusion, tiotropium appears to improve lung function and reduce the need for rescue treatments in adolescents with severe asthma and is well tolerated.

### Evidence of Tiotropium Bromide Use in School-Aged Children With Asthma

Once demonstrated that tiotropium was to be well tolerated and efficacious in adolescents with uncontrolled asthma, it became urgent to assessing its use for treatment of younger children. There are fewer therapeutic options for school-age children than adolescents and adults, however, this age group shows more unmet medical needs than others. A systematic review on the efficacy and safety of tiotropium as an add-on in children with moderate to severe asthma lasting from >3 to >6 months, uncontrolled despite use of an ICS with or without additional controller medications (32), has included the analysis of 3 RCT of 905 children aged 6–11 years (33–35). Duration of treatment ranged from 12- to 48-weeks. Once-daily tiotropium (5, 2.5, or 1.25 μg) improved lung function parameters, including peak and trough FEV1 or morning and evening PEF (only 5 μg dose) vs. placebo, however there were no statistically significant differences in asthma control and quality of life (33). Huang and coworkers conducted a 12 week-study of eighty children aged 6–14 years, with newly diagnosed moderate persistent asthma who were randomly administered fluticasone propionate aerosol or fluticasone propionate aerosol plus tiotropium *via* the dry powder HandiHaler^®^ device (36). They showed that lung function significantly improved in both groups at 4, 8, and 12 weeks compared with baseline, in particular in the tiotropium group compared to the control group. Of all clinical variables, no significant difference in the incidence of severe asthma between the two groups (36.3 and 26.8%, respectively) was found, however the proportion of days and frequency of SABA use and awakenings during the night was significantly reduced in children given tiotropium, with no severe adverse effects (36).

In conclusion, in school-aged children, the use of tiotropium appears to improve lung function and, albeit on limited data, reduces the risk of exacerbations and the need for corticosteroid therapy even though a significant difference in the incidence of severe asthma was not documented.

### Evidence of Tiotropium Bromide Use in Pre-school-Aged Children With Asthma

In early life, asthma presentation and clinical course are very different from those described in school-aged children and adolescents due to variable phenotypic heterogeneity and different responses to asthma medications (37). Yet asthma treatment of preschool children is challenging since a high proportion of patients who require frequent health care use because of asthma exacerbations belong to that age group, and this makes prevention of such events a crucial goal for reducing future morbidity (38). However, in young children the response to ICS is sometimes unpredictable because of different airway inflammatory findings (39). Yet not all young children have evidence of eosinophilic airway inflammation, even those with recurrent severe multi-trigger wheeze, and this can justify the poor response to ICS at least in selected cases (40). All the above issues explain why alternative therapeutic options to ICS are warranted in the preschool age group.

In a small exploratory RCT of limited duration (only 12 weeks), tiotropium bromide was administered at 5 and 2.5 μg to 102 children aged 1–5 years with persistent asthma compared to a placebo group (41). The study showed no benefit in the primary outcome measures, i.e., safety, assessed by comparing adverse events between the active and placebo groups, and efficacy, measured as the change in weekly mean combined daytime asthma symptom score from baseline to week 12. Adverse events were less frequent with tiotropium treatment than with placebo, however, no formal statistical comparison between groups was performed by the authors, and more importantly, no significant differences in symptom scores vs. the placebo group were found (41).

A very recent study conducted by Zielen and coworkers in children aged <6 years with uncontrolled severe asthma has showed that adding tiotropium bromide to LABA/ICS significantly improved the systemic corticosteroid prescriptions, the physician’s visits and the antibiotics need recorded 6 months before and after treatment (42). However, the study design, that included the analysis of electronic records, has indeed many limitations, primarily its retrospective nature and the extremely low number of patients enrolled. An ongoing open-label trial of infants and toddlers with recurrent episodes of wheeze and/or shortness of breath is evaluating the effects on episode-free days of a novel strategy of LAMA administration, i.e., as needed intermittent inhaled tiotropium bromide (5 μg once a day, beginning at the onset of an upper respiratory tract infection and continuing for 7–14 days) and as needed SABA vs. intermittent fluticasone propionate and SABA as needed, or solely SABA as needed (NCT03199976). The rationale of the study is that in young children viral-induced wheezing episodes are associated with increased parasympathetic nerve activity, therefore acetylcholine production can be blocked by the inhaled anticholinergic agent tiotropium (43). In conclusion, based on the findings of the scant literature on tiotropium bromide at preschool age, at present there is insufficient evidence to support efficacy of tiotropium use in infants and toddlers with persistent asthmatic symptoms.

## Eligibility Criteria and Doses

Based on the evidence from literature data on asthma (44), indications for administration of tiotropium bromide inhalation spray include the long-term, once-daily, maintenance treatment of moderate-to-severe asthma that is not adequately controlled on ICS. The drug was approved by the US Food and Drug Administration in 2015 in patients with asthma aged ≥12 years, and more recently in February 2017 in pediatric patients aged ≥6 years (18). The approved doses are 2.5 μg in the United States and 5 μg in the European Union (45).

## Future Needs

The goal of asthma management is to achieve symptom control and prevent exacerbations by prescribing a therapeutic plan which ensures the greatest clinical benefits and the smallest risk of adverse effects to the patients. Current treatment options for children and adolescents with asthma are progressively growing, and overall, most bronchodilators and anti-inflammatory medications are effective on relevant clinical and lung function outcomes.

Ideally, as low adherence to multiple daily treatments is a big issue in school-aged children and adolescents, providing antiasthma medications once-daily *via* an easy-to-use inhaler has a beneficial added value.

A major issue that should not be underestimated when antiasthma treatment is prescribed to young children is lack of cooperation in inhaling medications (46). In young children the preferred delivery system of inhaled medications is the pressurized metered-dose inhaler with a valved spacer (with or without a face mask, according to the patient’s age) (9). Future research also including the development of devices designed for different pediatric patients ages and sizes will hopefully improve the standard of care to infants and children with severe wheezing disorders (46).

FEV_1_ is good indicator to assess the severity of asthma or the efficacy of asthma medications in adult population studies about tiotropium. However, FEV_1_ may not be the best measure of outcome of pediatric asthma because children spirometry does not always correlate well with symptom severity, especially during asthma exacerbations (47). Thus, since a *post-hoc* analysis found that improvements in FEF25–75% response with tiotropium vs. placebo were largely more pronounced than improvements in FEV_1_ (47), measurement of low to medium lung volume flows may be a more sensitive than FEV_1_ for evaluating peripheral airway response to tiotropium in children and adolescents.

Although studies of tiotropium in children and adolescents overall show improvement of spirometry, the small sample sizes, and short study duration of the trials indicate that the impact of tiotropium should be investigated in longer-term trial cohorts of sufficient size to estimate the maximum clinical effect and the long-term safety (48). Lung function should not be the single endpoint of future studies. Indeed, most of the RCTs demonstrate that spirometry significantly improved, but subjective clinical symptoms evaluated by the asthma questionnaires, or the proportion of exacerbations modestly or not significantly improved (29, 30, 33, 41). Finally, also quality of life should be included as a substantial outcomes measure. In conclusion, pediatric research on tiotropium needs to be indeed directed toward several primary objectives possibly including the identification of predictor response. Future studies should focus on the identification of subgroups of children or adolescents with severe asthma preferentially responsive to LAMA who do not show beneficial effects from treatment with ICS/LABA or high ICS dose. Interestingly, patients with fixed or baseline airflow obstruction might preferentially respond to LAMA, as indicated by two trials in children and adolescents that enrolled asthma patients with FEV1 60–90% predicted (30, 34). In addition to this, other clinical outcome measures, for instance a high proportion of exacerbations or worse asthma control test scores at baseline should be considered in the study design to identify which patients respond better than others to treatment. In the phase III RCT of a large group of children and adolescents, Szefler and coworkers concluded that the effects of tiotropium bromide as an add-on treatment were not influenced by Th 2 phenotype, indicating that the decision of adding tiotropium does not require the evaluation of Th 2 and that tiotropium is effective regardless of allergic status (49). Based on these findings, tiotropium bromide was proposed as alternate option to biologic agents which are recommended to patients aged ≥6 years with severe asthma allergic phenotype (50). Yet biologics are very expensive, therefore, pending further comparative studies of biologics vs. LAMA, tiotropium bromide may also be considered as an appropriate option to biologics in children or adolescents with uncontrolled severe asthma and a confirmed Th 2 phenotype.

Cost-utility of tiotropium bromide in children and adolescents has been rarely discussed. A unique study of children has shown that add-on tiotropium bromide achieves better outcomes at lower cost compared to ICS/LABA therapy (51).

Recently, the glycopyrronium and umeclidinium LAMA combined with LABA and ICS have been studied as add-on triple therapy in adults with asthma that is uncontrolled despite treatment with an ICS/LABA association (13). The single-inhaler ICS/LABA/LAMA regimen is now recommended by GINA before any biologic or systemic steroid treatment is initiated in individuals aged 18 years or older at GINA step 5 (9). There are no published pediatric studies of ICS/LABA/LAMA triple therapy and, given the beneficial effects on pulmonary function in adults (13), whether the regimen is efficacious also in children older than 6 years with uncontrolled asthma should be explored.

An additional point that deserves to be pointed out is the possible effects of tiotropium bromide on airways inflammation and remodeling. As several cells involved in the inflammatory cascade of the asthma process express muscarinic receptors, it has been hypothesized that tiotropium can modulate the function of these cells and attenuate airway inflammation and smooth muscle mass thickening (52). In an animal model of chronic asthma, Kistemaker et al. showed that eosinophilic inflammation in response to allergen exposure and remodeling were reduced by combined administration of tiotropium and ciclesonide (53), suggesting that inhibition of airway inflammation and remodeling may contribute to the long-term beneficial effects of tiotropium (54). An additional mechanism through which anticholinergic drugs may impact on airway diseases is by modulating the asthma-associated mucus overproduction. In a study of mice and mucin production *in vitro*, Arai et al. showed that tiotropium inhibits neutrophil elastase-induced goblet cell metaplasia, probably by suppressing inflammation and through a direct action on epithelial cells (55). These effects of tiotropium bromide have been poorly investigated in humans and might be the issue of future pediatric studies, also including the comparison vs. the cornerstone anti-inflammatory treatment of asthma with ICS.

## Conclusion

Tiotropium bromide is the only LAMA licensed for asthma long-term treatment of patients aged ≥6 years who continue to have symptoms despite controller medication administration. Since the greatest effects of tiotropium bromide on lung function was evaluated in the short-term, whether treatment could affect also the long-term evolution of lung function is unknown. Most relevant changes are reported in spirometry when patients are administered 5 μg rather than other doses. Clinical effects are less significant, probably because most pediatric protocols include a short treatment period and therefore could not appraise the maximum clinical effect of an add-on therapy. An important limitation is also the difference in treatment duration (12 or 48 weeks), which hampers establishment of the long-term effectiveness of the medication.

Treatment with tiotropium bromide as an add-on medication appears to be well tolerated by children and adolescents with suboptimal control of moderate-to-severe asthma, is safe and no fatal events have been reported so far. However, long-term safety should be evaluated in future studies including longer periods of treatment. Since the goal of asthma treatment is to minimize symptom burden and risk of exacerbations, achieving adequate control of asthma symptoms is also imperative for reducing the risk of development of severe asthma. Although the results from the studies of children and adolescents with moderate-to-severe asthma are promising, additional well powered trials are needed to further assess the safety and efficacy of tiotropium bromide added-on to long-term treatment in larger pediatric populations also including preschool children, a population with special needs in whom the novel as needed intermittent administration might be an ideal treatment strategy.

## Author Contributions

FS and MB made substantial contributions to conception and design, involved in drafting the manuscript, and gave final approval of the version to be published. CZ conceived the idea, involved in drafting the manuscript, and gave final approval of the version to be published. PL made substantial contributions to conception and design, involved in drafting the manuscript and revised it critically for important intellectual content, and gave final approval of the version to be published. CB as graduate in Pharmacy supported all co-authors in the final revision of the manuscript, focusing on the critical aspects of tiotropium use in children and adolescents, and gave approval of the version to be published. All authors contributed to the article and approved the submitted version.

## Conflict of Interest

The authors declare that the research was conducted in the absence of any commercial or financial relationships that could be construed as a potential conflict of interest.

## Publisher’s Note

All claims expressed in this article are solely those of the authors and do not necessarily represent those of their affiliated organizations, or those of the publisher, the editors and the reviewers. Any product that may be evaluated in this article, or claim that may be made by its manufacturer, is not guaranteed or endorsed by the publisher.
